# Supplementary material for: Trafficking dynamics of VEGFR1, VEGFR2, and NRP1 in human endothelial cells
Source: PLoS Comput Biol. 2024 Feb 7;20(2):e1011798. doi: 10.1371/journal.pcbi.1011798 (PMC10878527; doi:10.1371/journal.pcbi.1011798)
Supplement: S1 File — Additional description of methods, including the impact of cell geometry on equations and parameters, and estimation of the values of receptor dimerization rate constants. Includes sources for justification of key parameters [5, 6, 37–40]. (PDF) [file pcbi.1011798.s001.pdf]

S1 File. SUPPLEMENTAL METHODS

Impact of cell geometry on the equations and parameters.

The units of receptor density used in our equations is the number of receptors per cell (#/cell), which enables us to match the calculated receptor levels with the experimental measurements of these receptor densities, which involve the aggregation of protein from many cells. Most of the rate constants used in the model, and particularly those related to trafficking, are first order (units of inverse time, 1/sec), and thus are unaffected by the selection of units for receptor density. The units for the production rates of the receptors ((#/cell)/sec) are consistent with the units for receptor density.

Where careful consideration must be made is in the impact of cell geometry on the units of the rate constants for receptor dimerization, particularly the second-order coupling rate constant:

Uncoupling rate constant, first order; units of $k_d = s^{-1}$

Coupling rate constant, second order; units of $k_c = (\#/cell)^{-1}s^{-1}$

Equilibrium constant ($K_d = k_d/k_c$), units of #/cell

Using units of #/cell in the coupling rate does not correctly represent the local density of receptors for the purposes of dimerization interactions. For example, if there were 1000 receptors on the surface, and 1000 receptors in Rab4 endosomes, but the Rab4 endosomes had a combined surface area half that of the cell surface, then we would expect the local receptor density (e.g. in units of $\#/\mu m^2$) to be higher in the endosomes; the receptors there, being closer together, would be more likely to interact. Therefore, we adjust the local coupling rate constants, such that they are consistent in units of $(\#/\mu m^2)^{-1}s^{-1}$ and adjusted by compartment surface area when incorporated into the equations in units of $(\#/cell)^{-1}s^{-1}$. To convert from one unit to the other:

$$k_c\ [(\#/cell)^{-1}s^{-1}] = k_c\ [(\#/\mu m^2)^{-1}s^{-1}]\ /\ [(\text{compartment area in } \mu m^2)/cell]$$

If the respective surface areas of the various compartments are the same, then the local coupling rate constants will be the same; if not, then the coupling rate constants will take smaller values for larger-area compartments (reflecting the lower receptor density).

We have previously estimated that a reasonable plasma membrane area for the surface compartments of endothelial cells is 1000 μm^2. For the endosomes, using a typical radius of 35 nm per endosome [37] and estimating a total endosomal volume of ~15 fL, approximately 1.5% of the cell volume [38,39] we estimate there to be 83,000 endosomes per cell [40]. This results in a total endosomal surface area of ~1275 μm^2, which if divided 3:1 between Rab4/5-endosomes and Rab11 endosomes, results in 950 μm^2 and 325 μm^2 for the two simulated endosomal compartments, respectively (Table S1).

Note, again, that these surface area estimates/assumptions affect only the local VEGFR dimerization rate constants in our simulations, and not the trafficking rate constants nor the simulation predictions of receptor density (nor the experimental measurements of receptor density).

Estimating the values of VEGFR dimerization rate constants

To estimate the rate constants for dimerization, we formulate simplified equations without trafficking and assume that the dimerization is at equilibrium:

$$\frac{1}{2}k_{c,RR}[R]^2 = k_{d,RR}[RR]$$

$$[R]^2 = 2K_{d,RR}[RR]$$

Assuming that [R] + 2[RR] = R_T (i.e., the total number of receptors is the sum of receptor monomers in monomer and dimer form), then:

$$(R_T - 2[RR])(R_T - 2[RR]) = 2K_{d,RR}[RR]$$

$$4[RR]^2 - 4R_T[RR] + R_T^2 = 2K_{d,RR}[RR]$$

$$[RR]^2 - \left(R_T + K_{d,RR}/2\right)[RR] + R_T^2/4 = 0$$

And so

$$[RR] = {}^1/_2\left(\left(R_T + K_{d,RR}/2\right) - \sqrt{\left(R_T + K_{d,RR}/2\right)^2 - R_T^2}\right)$$

or

$$[RR] = {}^1/_2\left(\left(R_T + K_{d,RR}/2\right) - \sqrt{K_{d,RR}^2/4 + R_T K_{d,RR}}\right)$$

And thus the theoretical dimeric fraction is:

$$2[RR]/R_T = \left(1 + K_{d,RR}/2R_T\right) - \sqrt{\left(K_{d,RR}/2R_T\right)^2 + K_{d,RR}/R_T}$$

$$\textit{or}\ 2[RR]/R_T = \left(1 + K_{d,RR}/2R_T\right) - \sqrt{\left(K_{d,RR}/2R_T\right)\left(2 + K_{d,RR}/2R_T\right)}$$

As you can see in a graph of this function (**S1 Fig**), the expected dimeric fraction increases as the overall receptor density (R_T) increases, and as the receptor coupling rate increases (i.e., affinity increases or K_d value decreases).

One of the key points to note here is that if the dimerization level is different for different total receptor levels, and the receptors are differentially localized within the cell (i.e., present at different densities at different locations, which we know is the case), then *locally* the dimeric fraction would be expected to be different (this is *in addition* to the surface area differences noted earlier). When we run the full simulation for HUVECs (**S2 Fig**), we can see that this is indeed the case; mass action kinetics will be different due to the local levels being different in each subcellular location. Note that because the surface and internal levels of VEGFR2 are very similar, the dimeric fraction is similar in the two locations, whereas for VEGFR1, the higher internal levels result in higher dimeric fractions internally.

Note that the full simulation does not match exactly with the theoretical results above, because trafficking will move the various receptor complexes between the subcellular locations, lessening the differences between them as it takes time for association and dissociation to occur.

To get approximately 40% dimerization of the VEGFRs [5,6] (**S2 Fig**) in further simulations, we use base coupling rate constants of $8\text{x}10^{-4}$ 1/(#/μm^2)/s for VEGFR1 and $2\text{x}10^{-3}$ 1/(#/μm^2)/s for VEGFR2.; this translates to coupling rate constants on the surface of $8\text{x}10^{-7}$ (#/cell)$^{-1}$s^{-1} for VEGFR1 and $2\text{x}10^{-6}$ (#/cell)$^{-1}$s^{-1} for VEGFR2.

NRP1-VEGFR1 Coupling

If we simplify the coupling of VEGFR1 and NRP1 by first ignoring VEGFR1 and NRP1 dimerization dynamics and focusing only on the NRP1-VEGFR1 coupling/uncoupling processes:

$$k_{c,RN}[R][N] = k_{d,RN}[RN]$$

Assuming that [R] + [RN] = R_T and [N] + [RN] = N_T (total receptors is the sum of coupled and uncoupled receptors) then

$$(R_T - [RN])(N_T - [RN]) = K_{d,RN}[RN]$$

$$[RN]^2 - (R_T + N_T)[RN] + R_T N_T = K_{d,RN}[RN]$$

$$[RN]^2 - \left(R_T + N_T + K_{d,RN}\right)[RN] + R_T N_T = 0$$

And thus:

$$[RN] = {}^{1}\!/_{2}\left(\left(R_T + N_T + K_{d,RN}\right) - \sqrt{\left(R_T + N_T + K_{d,RN}\right)^2 - 4R_T N_T}\right)$$

The fraction of R in RN complexes is [RN]/R_T, and the fraction of N in RN complexes is [RN]/N_T.

Thus, the fraction of R or N in RN complexes would be expected to increase as the total levels of the other receptor partner increases (**S3 Fig**), though the exact function is different from the VEGFR homodimerization (previous section).

However, the R1-N1 situation is complicated further when we consider the full dimerization of VEGFR1 and the existence of R1-R1-N1 and N1-R1-R1-N1 complexes. This is in addition to the formation of R1-N1 complexes.

$$2k_{c,RN}[RR][N] = k_{d,RN}[RRN] \quad \text{and} \quad k_{c,RN}[RRN][N] = 2k_{d,RN}[NRRN]$$

The effective affinity ($K_{d,eff}$) of NRP binding to RR is then:

$$K_{d,eff} = \frac{[RR][N]}{[RRN] + 2[NRRN]} = \frac{[RRN]\,{}^{K_d}\!/_{2}}{[RRN] + [RRN][N]\,{}^{1}\!/_{K_d}}$$

$$K_{d,eff} = \frac{K_d/2}{1 + [N]\,{}^1/_{K_d}} = K_d \frac{1}{2\left(1 + {}^{[N]}/_{K_d}\right)}$$

As N increases, the effective affinity gets lower. Note that the N in this equation is N(t), i.e., the free (unbound) available NRP1 at the given time, not total NRP1 (N_T). Thus, the effective K_d not only changes by location but also with time as binding occurs. However, in the range of concentrations we are typically seeing in HUVECs (1-100 μm^{-2}), and for a typical $K_{d,RN}$ (50 μm^{-2}), $K_{d,eff}$ changes only a factor of three across two orders of magnitude of NRP expression (**S4 Fig**). Thus, the impact of using a constant k_c or constant K_d is small. Note that this effect is in addition to the total receptor density and K_d effects on dimerization noted above.

As with VEGFR dimerization, because the levels of both VEGFR1 and NRP1 are different at different locations in the cell, thus we would expect the fraction of each receptor involved in RN coupling to be different at each location, and indeed when we run the full simulation, that is what we see (**S5 Fig**). VEGFR1 is more commonly found in R1-N1 complexes on the surface than inside the cell, because NRP1 is in excess over VEGFR1 on the surface; while a higher proportion of NRP1 is associated with VEGFR1 in Rab4 endosomes than on the surface, because VEGFR1 is in excess over NRP1 there.

If we use a similar coupling rate constant for VEGFR1-NRP1 as for VEGFR1-VEGFR1 (i.e., $k_{c,RN} = k_{c,R1R1} = 0.0008\ (\#/\mu m^2)^{-1}.s^{-1}$), this results in approximately 20% of VEGFR1 in complex with NRP1 across the cell (~75% on the cell surface), and 4% of NRP1 in complex with VEGFR1 across the cell (~85% in Rab4 endosomes) (**S5 Fig**). We will use this coupling rate value as a starting point and explore the effect of different values.
