# Supplementary material for: Trafficking dynamics of VEGFR1, VEGFR2, and NRP1 in human endothelial cells
Source: PLoS Comput Biol. 2024 Feb 7;20(2):e1011798. doi: 10.1371/journal.pcbi.1011798 (PMC10878527; doi:10.1371/journal.pcbi.1011798)
Supplement: S1 Fig — Theoretical estimate of VEGFR dimerization, depending on coupling rate constant (kc) (A) and total receptor density (RT in #/cell) (B). Here the uncoupling rate constant, kd = 0.01 s-1, and thus for kc = 10−5 (#/cell)-1s-1, equilibrium constant (Kd = kd/kc) is 1000 #/cell and for kc = 10−7 (#/cell)-1s-1, Kd = 105 #/cell. (PDF) [file pcbi.1011798.s002.pdf]

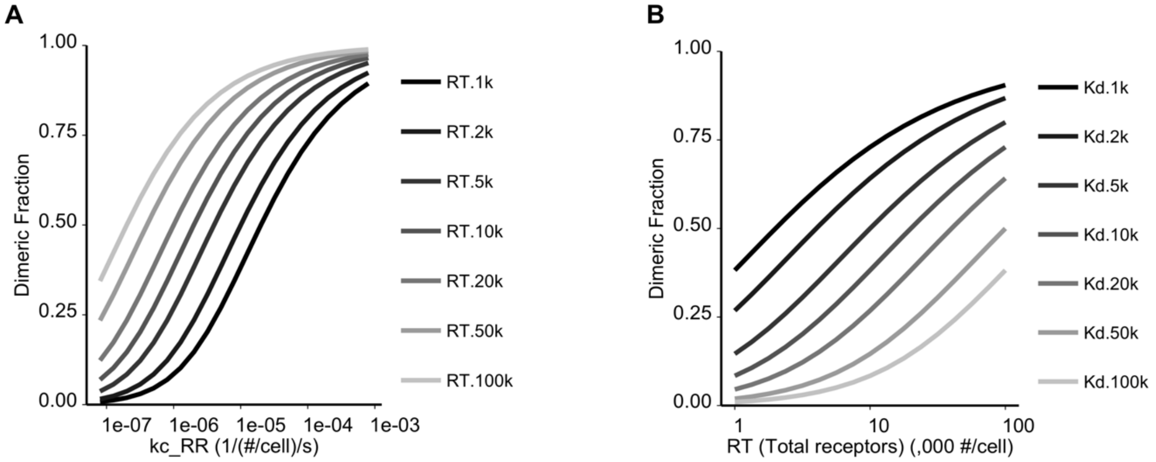

S1 Fig. Theoretical estimate of VEGFR dimerization, depending on coupling rate constant (k_c) **(A)** and total receptor density (R_T in #/cell) **(B)**. Here the uncoupling rate constant, k_d = 0.01 s^{-1}, and thus for $k_c = 10^{-5}$ $(\#/cell)^{-1}s^{-1}$, equilibrium constant ($K_d = k_d/k_c$) is 1000 #/cell and for $k_c = 10^{-7}$ $(\#/cell)^{-1}s^{-1}$, $K_d = 10^5$ #/cell.
