# Supplementary material for: Trafficking dynamics of VEGFR1, VEGFR2, and NRP1 in human endothelial cells
Source: PLoS Comput Biol. 2024 Feb 7;20(2):e1011798. doi: 10.1371/journal.pcbi.1011798 (PMC10878527; doi:10.1371/journal.pcbi.1011798)
Supplement: S2 Fig — Simulated dimerization of VEGFR1 (A) and VEGFR2 (B) on HUVECs, by subcellular location, depending on the underlying coupling rate constant. Note that the local coupling rate constant is then adjusted by surface area as noted above; for example, for a base coupling rate constant of 10−4 1/(#/μm2)/s, the local coupling rate at the surface would be 10−7 1/(#/cell)/s. (PDF) [file pcbi.1011798.s003.pdf]

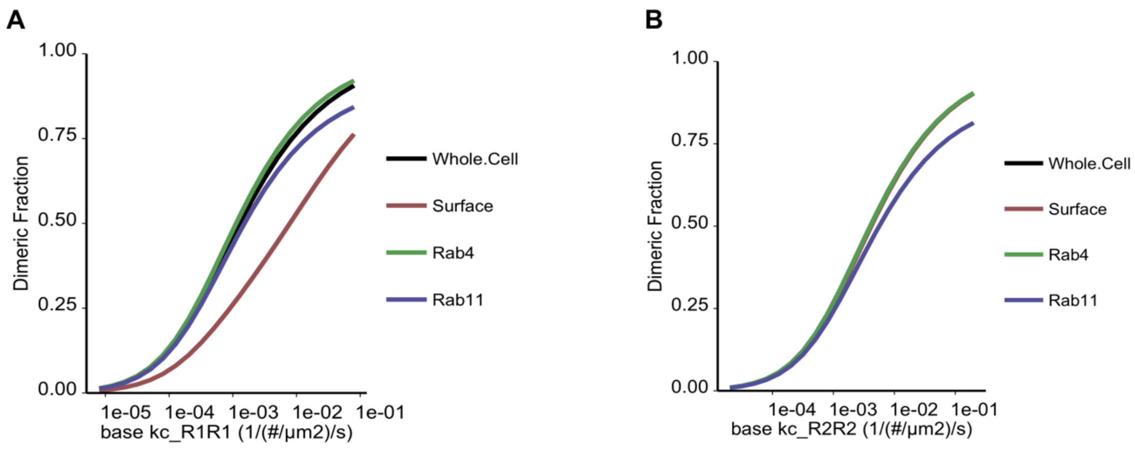

S2 Fig. Simulated dimerization of VEGFR1 (A) and VEGFR2 (B) on HUVECs, by subcellular location, depending on the underlying coupling rate constant. Note that the local coupling rate constant is then adjusted by surface area as noted above; for example, for a base coupling rate constant of 10^{-4} 1/(#/μm^2)/s, the local coupling rate at the surface would be 10^{-7} 1/(#/cell)/s.
