# Supplementary material for: Trafficking dynamics of VEGFR1, VEGFR2, and NRP1 in human endothelial cells
Source: PLoS Comput Biol. 2024 Feb 7;20(2):e1011798. doi: 10.1371/journal.pcbi.1011798 (PMC10878527; doi:10.1371/journal.pcbi.1011798)
Supplement: S3 Fig — Theoretical estimate of VEGFR1-NRP1 coupling, assuming 1:1 binding only, showing the fraction of all VEGFR1 (A) and all NRP1 (B) estimated to be in VEGFR1-NRP1 complexes, depending on total receptor densities (RT in #/cell; NT in #/cell). For this simulation, uncoupling rate constant kd = 0.01 s-1 and equilibrium constant Kd,RN = 12,500 #/cell. (PDF) [file pcbi.1011798.s004.pdf]

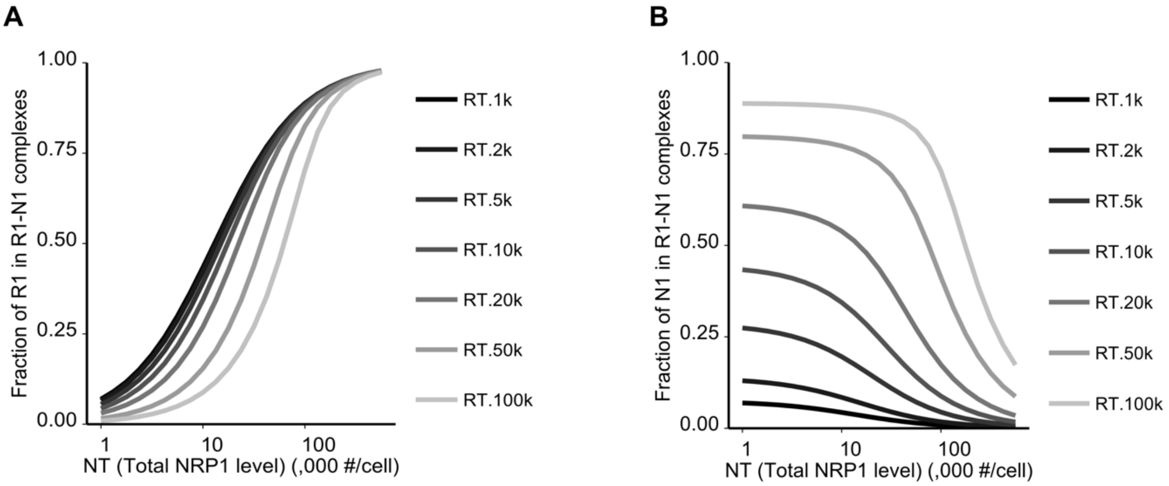

S3 Fig. Theoretical estimate of VEGFR1-NRP1 coupling, assuming 1:1 binding only, showing the fraction of all VEGFR1 **(A)** and all NRP1 **(B)** estimated to be in VEGFR1-NRP1 complexes, depending on total receptor densities (R_T in #/cell; N_T in #/cell). For this simulation, uncoupling rate constant $k_d = 0.01\ s^{-1}$ and equilibrium constant $K_{d,RN}$ = 12,500 #/cell.
