# Supplementary material for: Trafficking dynamics of VEGFR1, VEGFR2, and NRP1 in human endothelial cells
Source: PLoS Comput Biol. 2024 Feb 7;20(2):e1011798. doi: 10.1371/journal.pcbi.1011798 (PMC10878527; doi:10.1371/journal.pcbi.1011798)
Supplement: S4 Fig — (PDF) [file pcbi.1011798.s005.pdf]

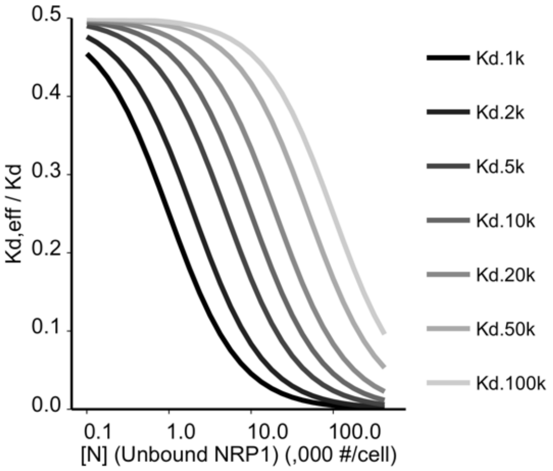

S4 Fig. Theoretical estimate of the effective affinity ($K_{d,eff}$) of NRP1 coupling to VEGFR1, assuming 2:2 binding, compared to the 1:1 binding affinity (K_d), as dependent on the local current unbound NRP1 levels and the 1:1 binding affinity.
