# Supplementary material for: Trafficking dynamics of VEGFR1, VEGFR2, and NRP1 in human endothelial cells
Source: PLoS Comput Biol. 2024 Feb 7;20(2):e1011798. doi: 10.1371/journal.pcbi.1011798 (PMC10878527; doi:10.1371/journal.pcbi.1011798)
Supplement: S5 Fig — Simulated dimerization of VEGFR1-NRP1, showing the fraction of all VEGFR1 (A) and all NRP1 (B) that are in VEGFR1-NRP1-containing complexes on HUVECs, by subcellular location, depending on coupling rate. (PDF) [file pcbi.1011798.s006.pdf]

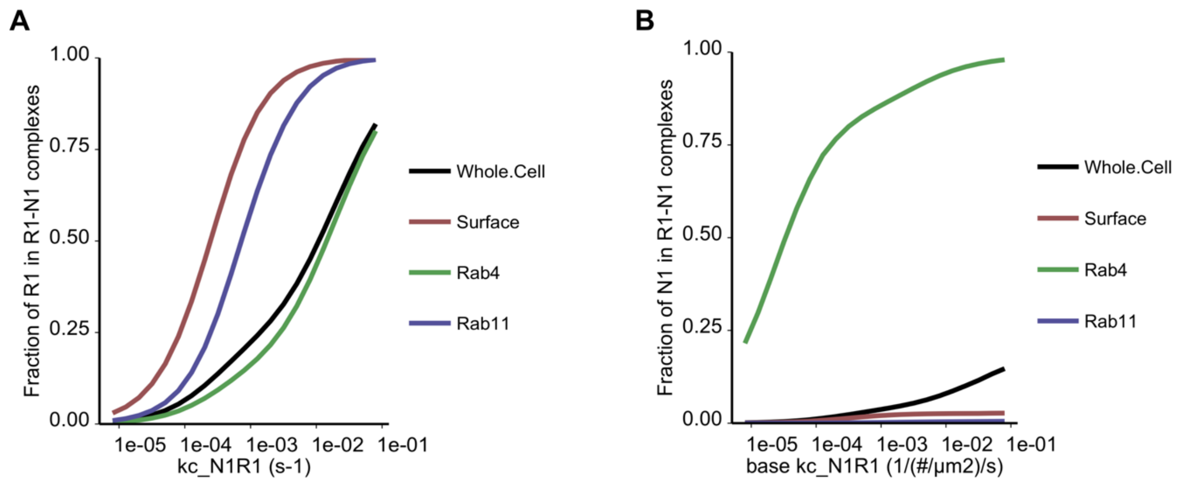

S5 Fig. Simulated dimerization of VEGFR1-NRP1, showing the fraction of all VEGFR1 **(A)** and all NRP1 **(B)** that are in VEGFR1-NRP1-containing complexes on HUVECs, by subcellular location, depending on coupling rate.
