# Supplementary material for: Trafficking dynamics of VEGFR1, VEGFR2, and NRP1 in human endothelial cells
Source: PLoS Comput Biol. 2024 Feb 7;20(2):e1011798. doi: 10.1371/journal.pcbi.1011798 (PMC10878527; doi:10.1371/journal.pcbi.1011798)
Supplement: S6 Fig — A, Control experiments to validate anti-VEGFR1 antibody. Western blot of HUVEC treated with siRNA against VEGFR1 (depleting both membrane-integral VEGFR1 (mFlt1) and soluble VEGFR1 (sFlt1)) or siRNA targeting mFlt1 or sFlt1 alone. B, Control experiments to validate the NRP1 antibody. Western blot of HUVEC treated with NRP1 siRNA or control siRNA. Reagents are detailed in S7 Table. (PDF) [file pcbi.1011798.s007.pdf]

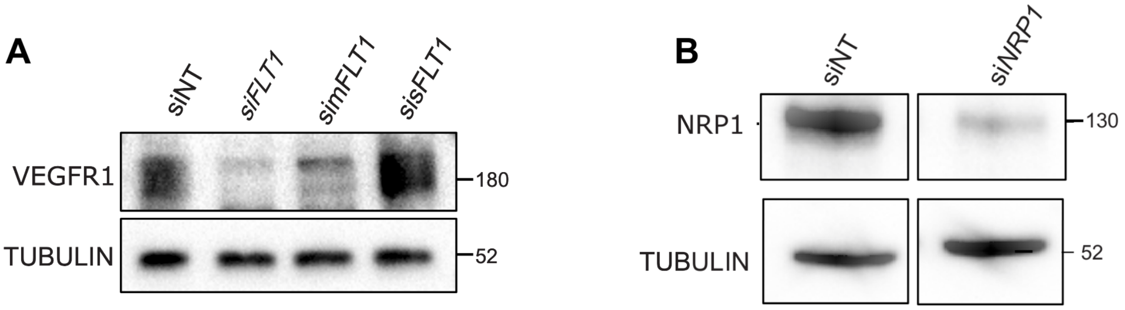

S6 Fig. Antibody validation. A, Control experiments to validate anti-VEGFR1 antibody. Western blot of HUVEC treated with siRNA against VEGFR1 (depleting both membrane-integral VEGFR1 (mFlt1) and soluble VEGFR1 (sFlt1)) or siRNA targeting mFlt1 or sFlt1 alone. **B,** Control experiments to validate the NRP1 antibody. Western blot of HUVEC treated with NRP1 siRNA or control siRNA. Reagents are detailed in S7 Table.
