# Supplementary material for: Trafficking dynamics of VEGFR1, VEGFR2, and NRP1 in human endothelial cells
Source: PLoS Comput Biol. 2024 Feb 7;20(2):e1011798. doi: 10.1371/journal.pcbi.1011798 (PMC10878527; doi:10.1371/journal.pcbi.1011798)
Supplement: S7 Fig — A, The schematics show how perturbations–cycloheximide (CHX) and the siRNA Rab knockdowns–are represented in the model. B-C, Changes in surface levels (B) and whole cell levels (C) of VEGFR1, VEGFR2, and NRP1 after Rab4a knockdown, Rab11a knockdown, and double Rab4a/Rab11a knockdown, compared to control (–, no siRNA treatment). These panels are similar to Fig 5E and 5F, but here the rate of coupling of VEGFR1 and NRP1 was set to zero. The dots in panel C represent experimental results (no change in whole cell VEGFR1, VEGFR2, NRP1 following knockdown treatment) (Fig 2C). (PDF) [file pcbi.1011798.s008.pdf]

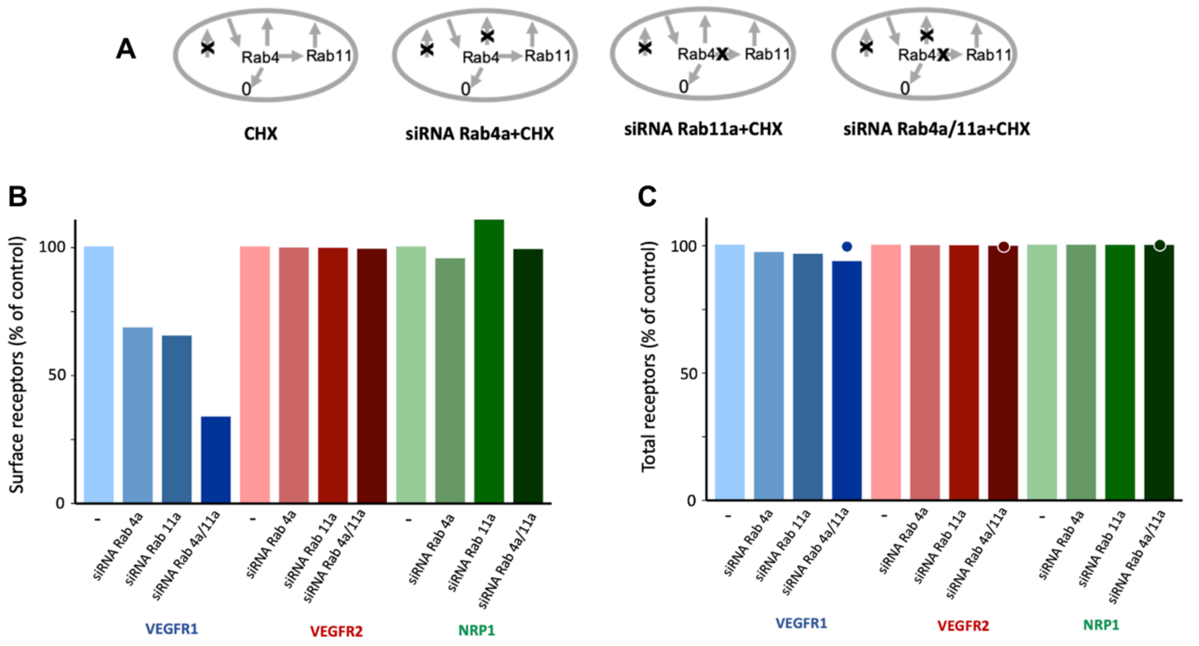

S7 Fig. Simulations of receptor level changes following Rab4a/11a knockdowns, in the absence of VEGFR1-NRP1 coupling. A, The schematics show how perturbations – cycloheximide (CHX) and the siRNA Rab knockdowns – are represented in the model. **B-C,** Changes in surface levels (B) and whole cell levels (C) of VEGFR1, VEGFR2, and NRP1 after Rab4a knockdown, Rab11a knockdown, and double Rab4a/Rab11a knockdown, compared to control (–, no siRNA treatment). These panels are similar to Figure 5E and 5F, but here the rate of coupling of VEGFR1 and NRP1 was set to zero. The dots in panel C represent experimental results (no change in whole cell VEGFR1, VEGFR2, NRP1 following knockdown treatment) (Fig 2C).
