# Supplementary material for: Trafficking dynamics of VEGFR1, VEGFR2, and NRP1 in human endothelial cells
Source: PLoS Comput Biol. 2024 Feb 7;20(2):e1011798. doi: 10.1371/journal.pcbi.1011798 (PMC10878527; doi:10.1371/journal.pcbi.1011798)
Supplement: S8 Fig — If we assume different values of the VEGFR1-NRP1 coupling rate constant (base rate of molecules-1.μm2.s-1; recall that this is adjusted to molecules-1.cell.s-1 at each location, as described in S1 File), and re-fit the parameters, only the NRP1 production rate parameter changes. Higher coupling requires higher NRP1 production, because the VEGFR1-NRP1 complex is lost at a faster rate than NRP1 alone. No further changes to any other optimized parameters (S6 Table) were needed to match the experimental data. (PDF) [file pcbi.1011798.s009.pdf]

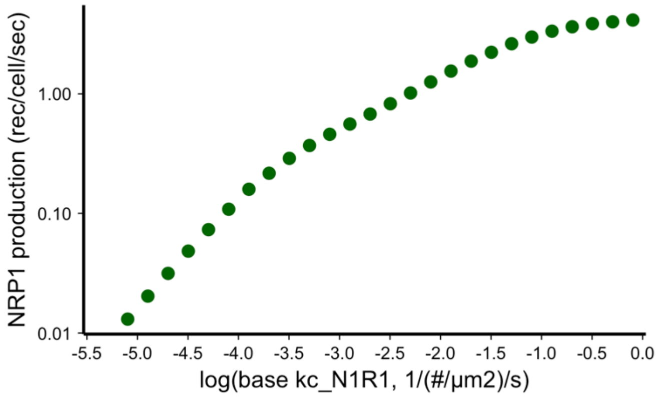


**S8 Fig. Neuropilin production rate depends on the VEGFR1-NRP1 coupling rate constant.** If we assume different values of the VEGFR1-NRP1 coupling rate constant (base rate of molecules$^{-1}$.µm$^{2}$.s$^{-1}$; recall that this is adjusted to molecules$^{-1}$.cell.s$^{-1}$ at each location, as described in S1 File**)**, and re-fit the parameters, only the NRP1 production rate parameter changes. Higher coupling requires higher NRP1 production, because the VEGFR1-NRP1 complex is lost at a faster rate than NRP1 alone. No further changes to any other optimized parameters (S6 Table) were needed to match the experimental data.
