# Supplementary material for: Trafficking dynamics of VEGFR1, VEGFR2, and NRP1 in human endothelial cells
Source: PLoS Comput Biol. 2024 Feb 7;20(2):e1011798. doi: 10.1371/journal.pcbi.1011798 (PMC10878527; doi:10.1371/journal.pcbi.1011798)
Supplement: S9 Fig — A, Whole-cell NRP1 levels are different before (filled circles) and following (empty circles) siRNA Rab knockdown; and these levels are different for different levels of VEGFR1-NRP1 coupling rates. B, The ratio of post-Rab-knockdown to pre-knockdown levels demonstrates the dependence of this ratio on the coupling rate. (PDF) [file pcbi.1011798.s010.pdf]

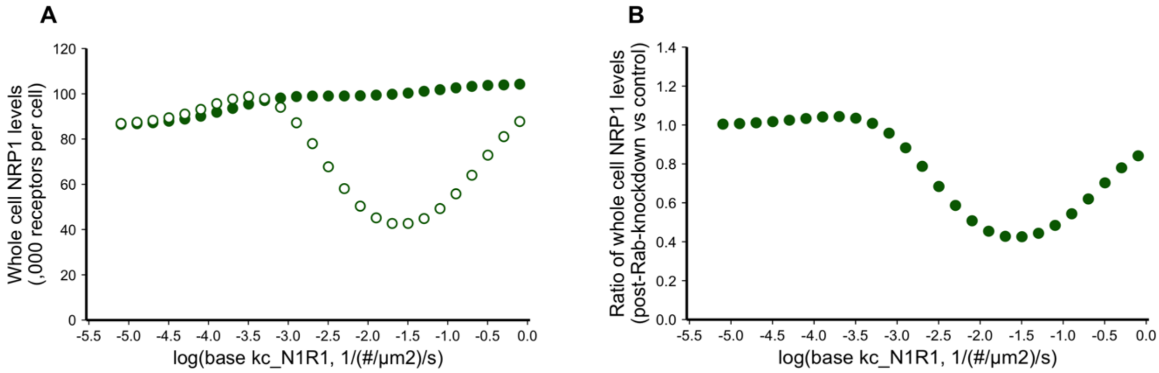

S9 Fig. Simulation-predicted levels of whole cell Neuropilin-1 (NRP1) levels under dual Rab4a/Rab11a knockdown treatment. A, Whole-cell NRP1 levels are different before (filled circles) and following (empty circles) siRNA Rab knockdown; and these levels are different for different levels of VEGFR1-NRP1 coupling rates. **B**, The ratio of post-Rab-knockdown to pre-knockdown levels demonstrates the dependence of this ratio on the coupling rate.
