# Supplementary material for: Trafficking dynamics of VEGFR1, VEGFR2, and NRP1 in human endothelial cells
Source: PLoS Comput Biol. 2024 Feb 7;20(2):e1011798. doi: 10.1371/journal.pcbi.1011798 (PMC10878527; doi:10.1371/journal.pcbi.1011798)
Supplement: S10 Fig — Local sensitivity analysis was performed by examining the sensitivity of model outputs to small changes in each of the receptor trafficking, degradation, and production parameters (S6 Table). Sensitivity values are the ratio of percent change in key model outputs (x-axis) to percent change in the parameter values (y-axis). This is similar to Fig 6, but here the rate of coupling of VEGFR1 and NRP1 was set to zero. (PDF) [file pcbi.1011798.s011.pdf]

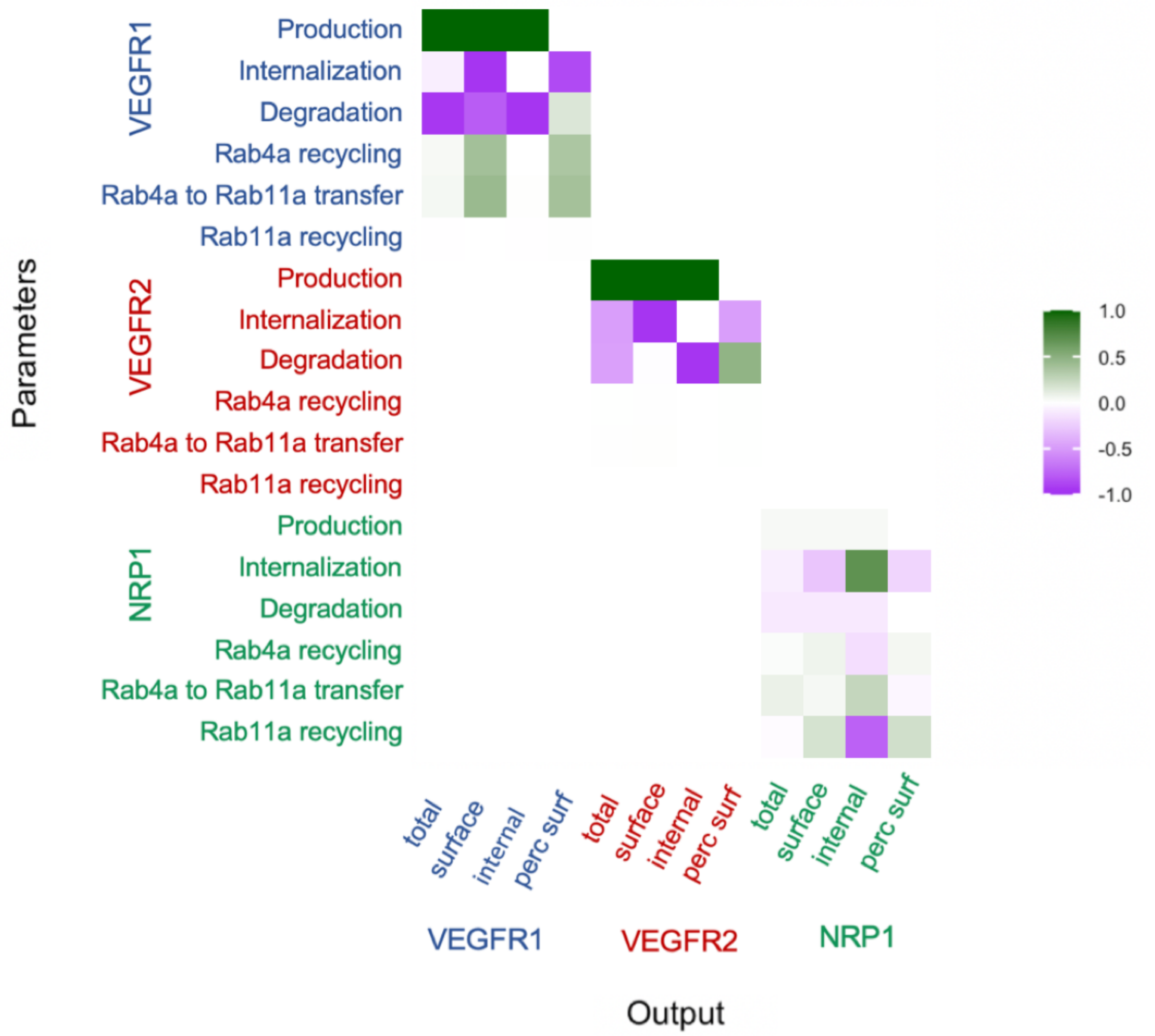

S10 Fig. Sensitivity of model outputs to VEGFR1, VEGFR2 and NRP1 trafficking parameters, in the absence of VEGFR1-NRP1 coupling. Local sensitivity analysis was performed by examining the sensitivity of model outputs to small changes in each of the receptor trafficking, degradation, and production parameters (S6 Table). Sensitivity values are the ratio of percent change in key model outputs (x-axis) to percent change in the parameter values (y-axis). This is similar to Fig 6, but here the rate of coupling of VEGFR1 and NRP1 was set to zero.
