# Supplementary material for: Trafficking dynamics of VEGFR1, VEGFR2, and NRP1 in human endothelial cells
Source: PLoS Comput Biol. 2024 Feb 7;20(2):e1011798. doi: 10.1371/journal.pcbi.1011798 (PMC10878527; doi:10.1371/journal.pcbi.1011798)
Supplement: S11 Fig — This panel shows how the model fit to experimental data changes as the parameter values change; in other words, it shows the contribution of each parameter to the overall cost. The cost function values are normalized to the lowest value across all global simulations and then the log is taken, thus the lowest cost is represented here as zero. (PDF) [file pcbi.1011798.s012.pdf]

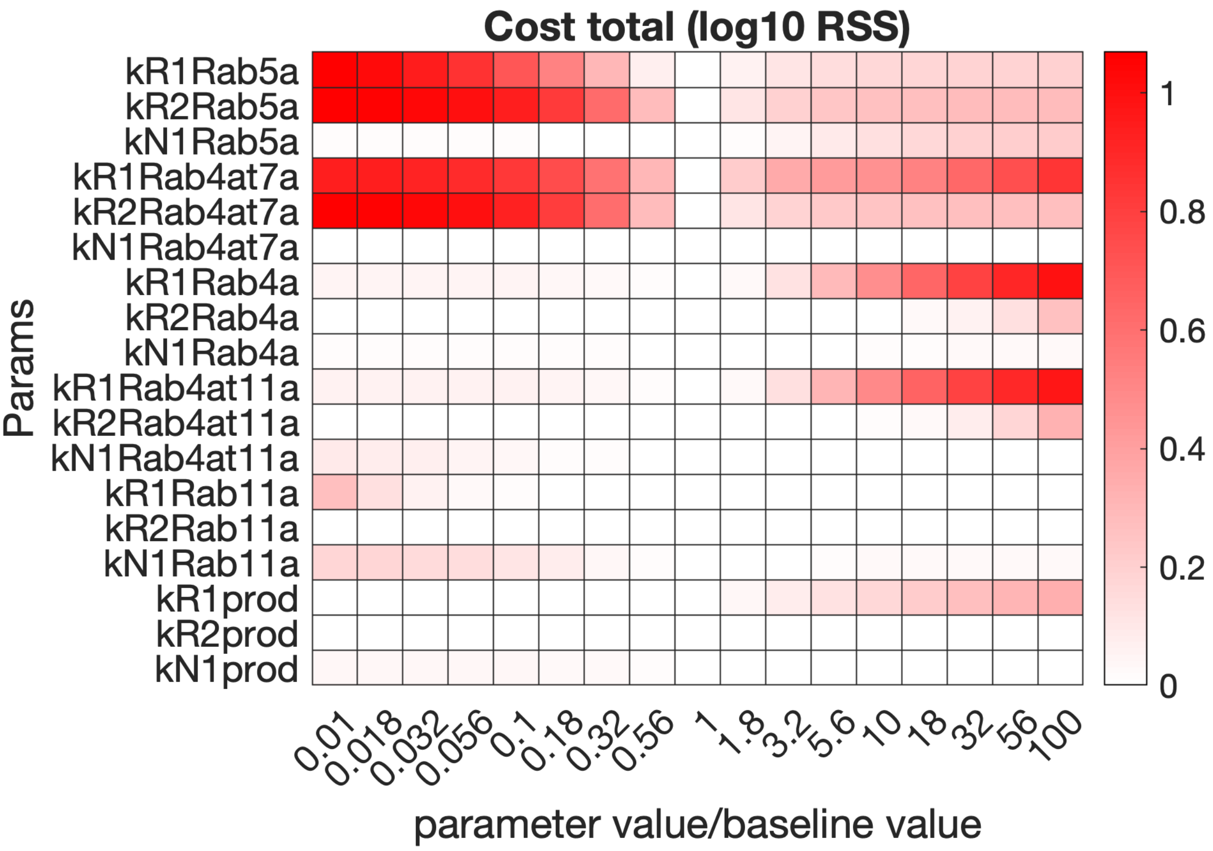

S11 Fig. Global sensitivity analysis – goodness of fit. This panel shows how the model fit to experimental data changes as the parameter values change; in other words, it shows the contribution of each parameter to the overall cost. The cost function values are normalized to the lowest value across all global simulations and then the log is taken, thus the lowest cost is represented here as zero.
