# Supplementary material for: Trafficking dynamics of VEGFR1, VEGFR2, and NRP1 in human endothelial cells
Source: PLoS Comput Biol. 2024 Feb 7;20(2):e1011798. doi: 10.1371/journal.pcbi.1011798 (PMC10878527; doi:10.1371/journal.pcbi.1011798)
Supplement: S13 Fig — This panel shows how changing the various trafficking and production parameters impacts the simulation predictions to be compared two key experimental data points: the absolute number of surface VEGFR2 (B) and the percentage of cell VEGFR2 that is on the surface (A). (PDF) [file pcbi.1011798.s014.pdf]

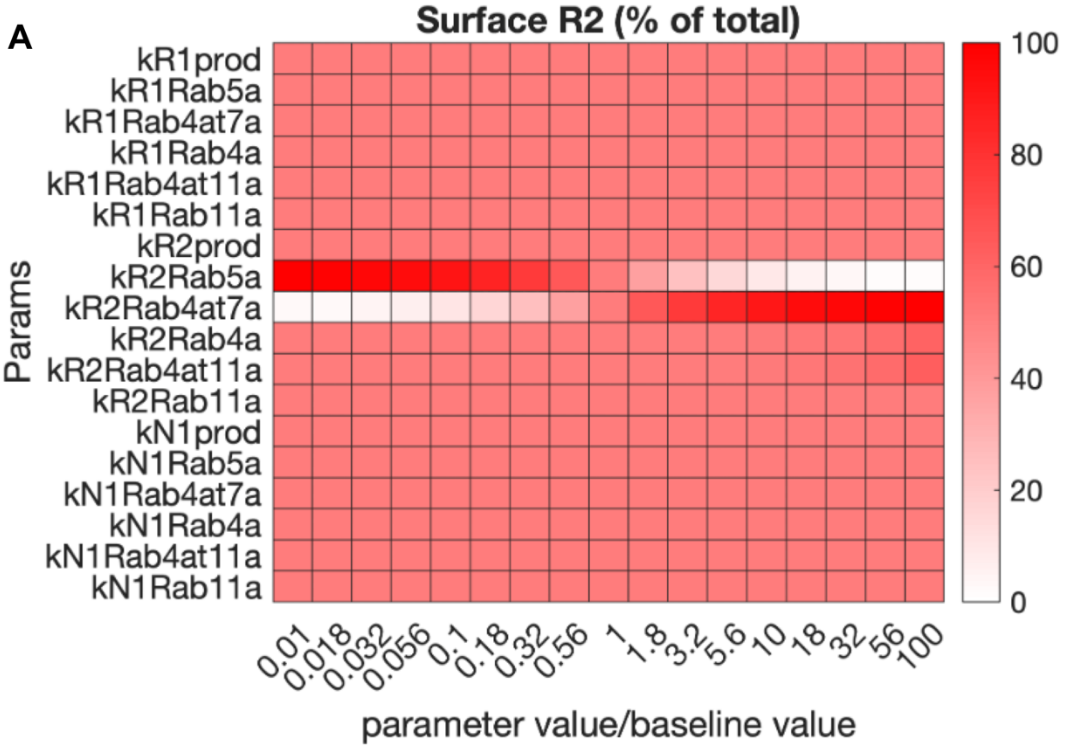

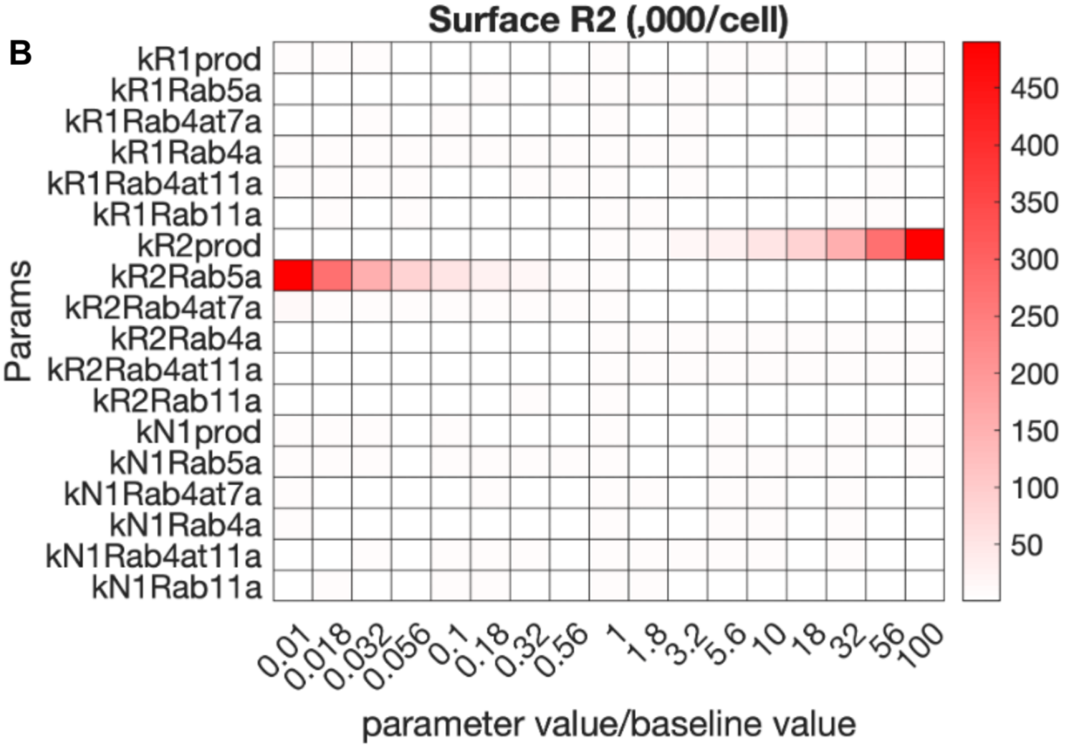

S13 Fig. Global sensitivity analysis – VEGFR2 on the surface. This panel shows how changing the various trafficking and production parameters impacts the simulation predictions to be compared two key experimental data points: the absolute number of surface VEGFR2 (**B**) and the percentage of cell VEGFR2 that is on the surface (**A**).
