# Supplementary material for: Trafficking dynamics of VEGFR1, VEGFR2, and NRP1 in human endothelial cells
Source: PLoS Comput Biol. 2024 Feb 7;20(2):e1011798. doi: 10.1371/journal.pcbi.1011798 (PMC10878527; doi:10.1371/journal.pcbi.1011798)
Supplement: S17 Fig — This panel shows how changing the various trafficking and production parameters impacts the simulation predictions to be compared two key experimental data points: the change in whole cell NRP1 two hours after CHX administration (A) and the change in whole cell NRP1 18 hours after administration of siRNA against Rab4a and Rab11a (B). (PDF) [file pcbi.1011798.s018.pdf]

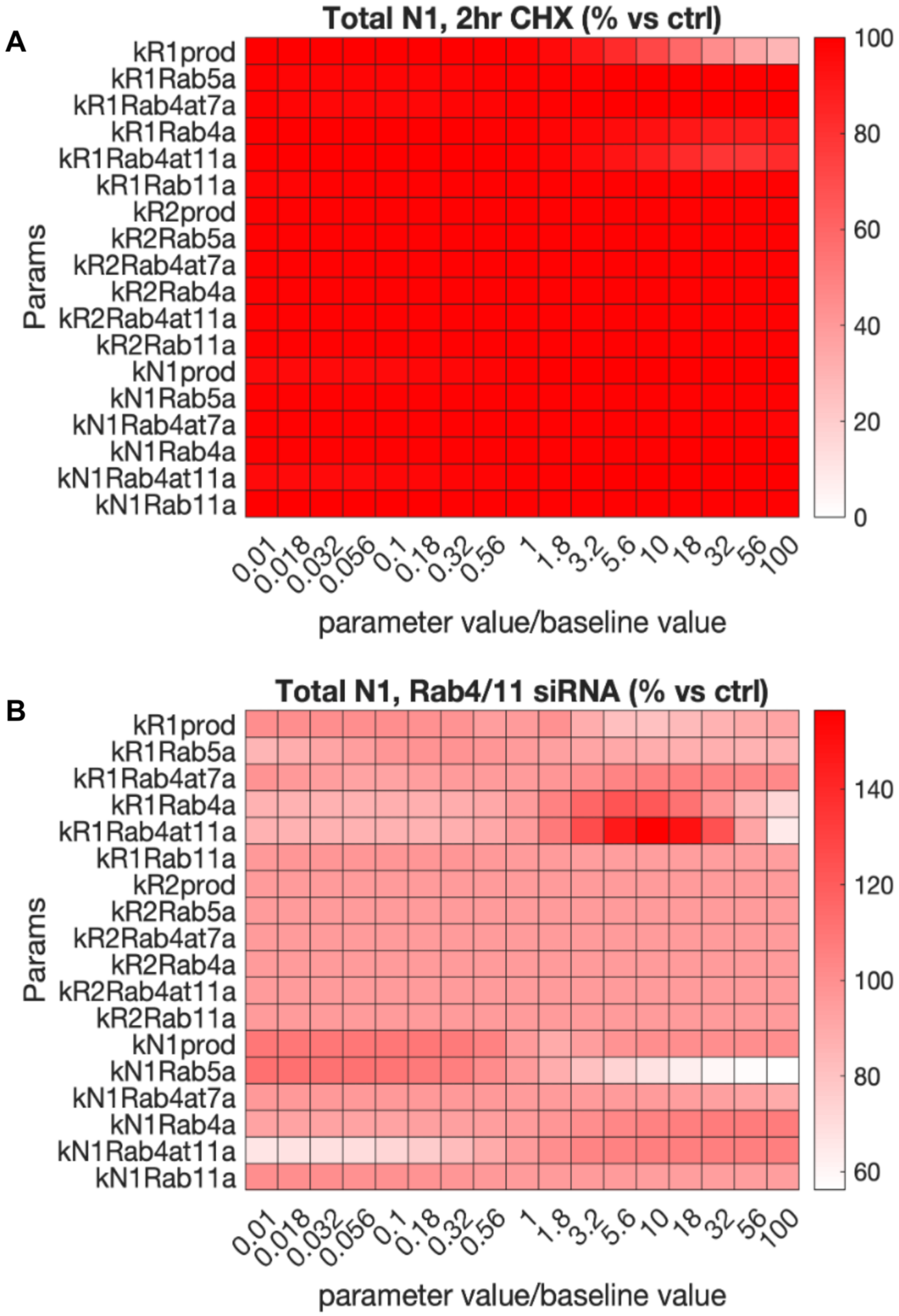

S17 Fig. Global sensitivity analysis – NRP1 following perturbations. This panel shows how changing the various trafficking and production parameters impacts the simulation predictions to be compared two key experimental data points: the change in whole cell NRP1 two hours after CHX administration (**A**) and the change in whole cell NRP1 18 hours after administration of siRNA against Rab4a and Rab11a (**B**).
