# Supplementary material for: Trafficking dynamics of VEGFR1, VEGFR2, and NRP1 in human endothelial cells
Source: PLoS Comput Biol. 2024 Feb 7;20(2):e1011798. doi: 10.1371/journal.pcbi.1011798 (PMC10878527; doi:10.1371/journal.pcbi.1011798)
Supplement: S18 Fig — Optimization of the trafficking parameters begins with initial guesses for the fifteen trafficking and degradation parameters. Each dot in the graphs above represents one of the 100 different optimized parameter sets. The production rates (the other three of the 18 optimized parameters) are continually optimized in an inner loop within the trafficking optimization loop, so they are not represented here as there is no single initial guess for those. If parameters were difficult for the optimization methodology to identify, we would see strong diagonal patterns in the graphs. Tight horizontal patterns represent parameters that are not only identifiable but also very highly constrained. The low correlation metrics between the initial and optimized parameter values are given in S8 Table. (PDF) [file pcbi.1011798.s019.pdf]

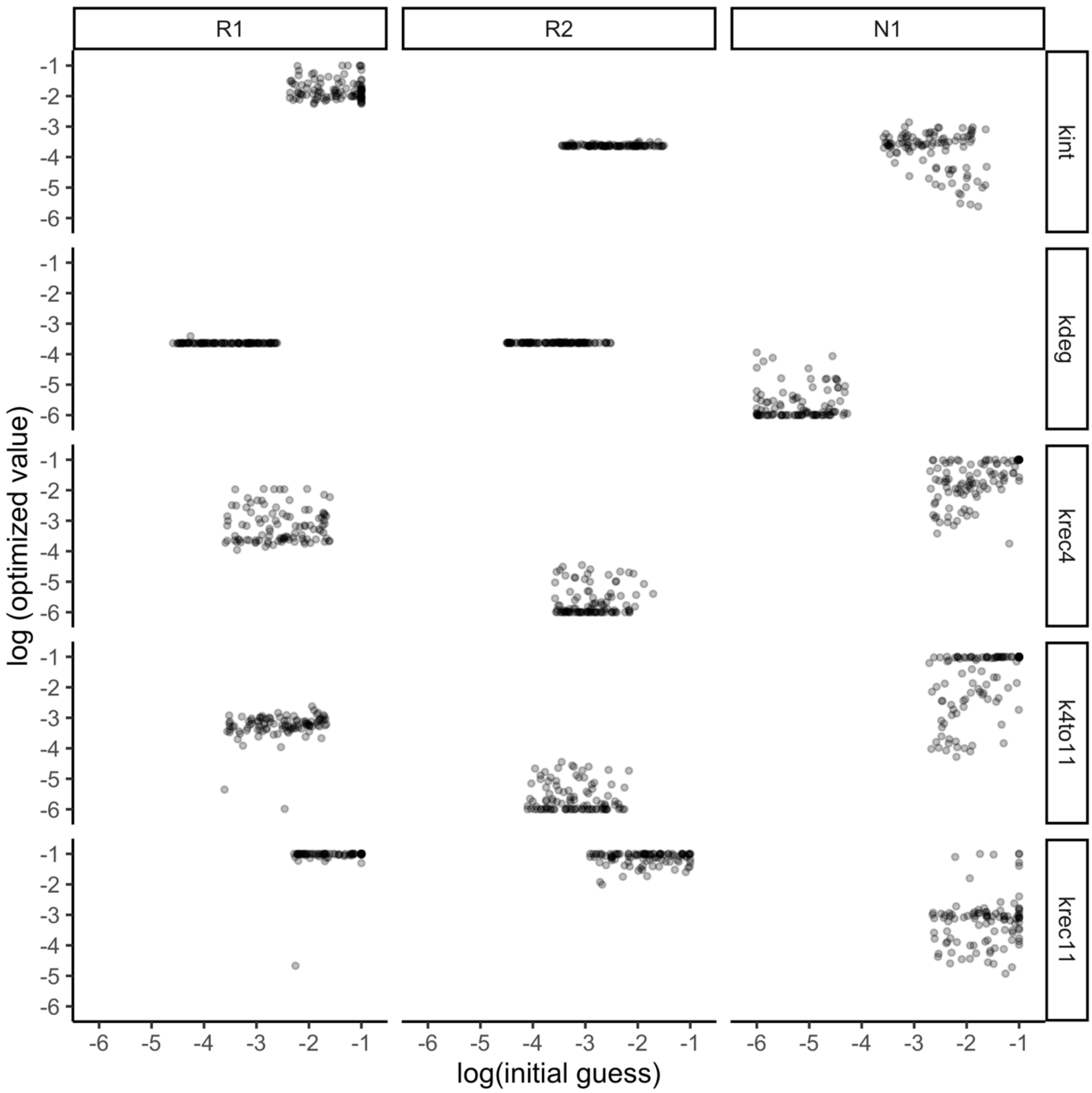

S18 Fig. Correlations between initial parameter guesses (x-axes) and optimized parameter values (y-axes). Optimization of the trafficking parameters begins with initial guesses for the fifteen trafficking and degradation parameters. Each dot in the graphs above represents one of the 100 different optimized parameter sets. The production rates (the other three of the 18 optimized parameters) are continually optimized in an inner loop within the trafficking optimization loop, so they are not represented here as there is no single initial guess for those. If parameters were difficult for the optimization methodology to identify, we would see strong diagonal patterns in the graphs. Tight horizontal patterns represent parameters that are not only identifiable but also very highly constrained. The low correlation metrics between the initial and optimized parameter values are given in S8 Table.
