# Supplementary material for: Trafficking dynamics of VEGFR1, VEGFR2, and NRP1 in human endothelial cells
Source: PLoS Comput Biol. 2024 Feb 7;20(2):e1011798. doi: 10.1371/journal.pcbi.1011798 (PMC10878527; doi:10.1371/journal.pcbi.1011798)
Supplement: S1 Table — There are 85 total individual reactions in the model: 3 for production of new receptors; 40 for four trafficking processes and one degradation process each applying to eight molecules (S6 Table); 21 coupling reactions (seven reactions in three locations) and 21 uncoupling reactions (seven reactions in three locations). For the coupling and uncoupling reactions, we assume that the VEGFR1-VEGFR1 and VEGFR1-NRP1 interactions are independent, and thus use the same coupling/uncoupling parameters for the various interactions. Note: the first three coupling reactions are explicitly coded as binding patterns in the model BioNetGen code, and BioNetGen then generates the four remaining coupling reactions so that all seven are present in the model MATLAB code. (PDF) [file pcbi.1011798.s020.pdf]

S1 Table. Model reactions. There are 85 total individual reactions in the model: 3 for production of new receptors; 40 for four trafficking processes and one degradation process each applying to eight molecules (S6 Table); 21 coupling reactions (seven reactions in three locations) and 21 uncoupling reactions (seven reactions in three locations). For the coupling and uncoupling reactions, we assume that the VEGFR1-VEGFR1 and VEGFR1-NRP1 interactions are independent, and thus use the same coupling/uncoupling parameters for the various interactions. Note: the first three coupling reactions are explicitly coded as binding patterns in the model BioNetGen code, and BioNetGen then generates the four remaining coupling reactions so that all seven are present in the model MATLAB code.

Category	Reaction examples	Notes
**Coupling and uncoupling reactions (second order forward, first order reverse)**		
	VEGFR1 + VEGFR1 <=> VEGFR1.VEGFR1<br>VEGFR2 + VEGFR2 <=> VEGFR2.VEGFR2<br>VEGFR1 + NRP1 <=> VEGFR1.NRP1<br><br>VEGFR1.NRP1 + VEGFR1 <=> VEGFR1.VEGFR1.NRP1<br>VEGFR1.NRP1 + VEGFR1.NRP1 <=> NRP1.VEGFR1.VEGFR1.NRP1<br>VEGFR1.VEGFR1 + VEGFR1 <=> VEGFR1.VEGFR1.NRP1<br>VEGFR1.VEGFR1.NRP1 + NRP1 <=> NRP1.VEGFR1.VEGFR1.NRP1	these reactions are repeated for each of 3 subcellular locations
**Trafficking and degradation reactions (first order)**		
Internalization<br>Recycling<br>Transfer<br>Recycling<br>Degradation	$VEGFR1_{surface}$ => $VEGFR1_{Rab4}$<br>$VEGFR1_{Rab4}$ => $VEGFR1_{surface}$<br>$VEGFR1_{Rab4}$ => $VEGFR1_{Rab11}$<br>$VEGFR1_{Rab11}$ => $VEGFR1_{surface}$<br>$VEGFR1_{Rab4}$ => $VEGFR1_{degraded}$	these reactions are repeated for each of the 8 molecules in Table S2
**Production (zeroth order)**		
	Ø => $VEGFR1_{surface}$<br>Ø => $VEGFR2_{surface}$<br>Ø => $NRP1_{surface}$	
