# Supplementary material for: Trafficking dynamics of VEGFR1, VEGFR2, and NRP1 in human endothelial cells
Source: PLoS Comput Biol. 2024 Feb 7;20(2):e1011798. doi: 10.1371/journal.pcbi.1011798 (PMC10878527; doi:10.1371/journal.pcbi.1011798)
Supplement: S2 Table — List of the 32 molecules and molecular complexes whose levels are predicted by the 32 ordinary differential equations of the mechanistic computational model. The numbers in the table refer to equation number and the number that represents that molecule in the in the model code (they appear non-sequential because of the order in which BioNetGen generates the molecular complexes). The concentrations of molecules at the surface and in the Rab4a and Rab11a endosomes are in units of #/cell and represent the current receptor densities in those locations. In contrast, the degraded molecules do not represent current concentrations but rather cumulative degraded receptors. Trafficking of monomers and dimers is assumed to be the same, and molecular complexes of VEGFR1 and NRP1 are assumed to use VEGFR1 trafficking parameters (see Methods). (PDF) [file pcbi.1011798.s021.pdf]

S2 Table. Molecules and molecular complexes in the model. List of the 32 molecules and molecular complexes whose levels are predicted by the 32 ordinary differential equations of the mechanistic computational model. The numbers in the table refer to equation number and the number that represents that molecule in the in the model code (they appear non-sequential because of the order in which BioNetGen generates the molecular complexes). The concentrations of molecules at the surface and in the Rab4a and Rab11a endosomes are in units of #/cell and represent the current receptor densities in those locations. In contrast, the degraded molecules do not represent current concentrations but rather cumulative degraded receptors. Trafficking of monomers and dimers is assumed to be the same, and molecular complexes of VEGFR1 and NRP1 are assumed to use VEGFR1 trafficking parameters (see Methods).

Output species	Surface	Rab4a5a	Rab11a	Degraded	Trafficking Parameters
vegfr1	1	7	15	18	VEGFR1
vegfr2	2	8	16	19	VEGFR2
nrp1	3	9	17	20	NRP1
vegfr1.vegfr1	4	12	23	26	VEGFR1
vegfr2.vegfr2	5	13	24	27	VEGFR2
nrp1.vegfr1	6	14	25	28	VEGFR1
nrp1.vegfr1.vegfr1	10	21	29	31	VEGFR1
nrp1.vegfr1.vegfr1.nrp1	11	22	30	32	VEGFR1
