# Supplementary material for: Trafficking dynamics of VEGFR1, VEGFR2, and NRP1 in human endothelial cells
Source: PLoS Comput Biol. 2024 Feb 7;20(2):e1011798. doi: 10.1371/journal.pcbi.1011798 (PMC10878527; doi:10.1371/journal.pcbi.1011798)
Supplement: S3 Table — Note that these base parameters (in units of molecules-1.μm2.s-1) are adjusted to units of molecules-1.cell.s-1 at each location, using the appropriate membrane surface area (S4 Table), as described in S1 File. Includes sources for justification of key parameters from previous studies [5,6,16,26,34]. (PDF) [file pcbi.1011798.s022.pdf]

S3 Table. Receptor dimerization parameters.
Note that these base parameters (in units of molecules^{-1} .µm^{2}.s^{-1}) are adjusted to units of molecules^{-1}.cell.s^{-1} at each location, using the appropriate membrane surface area (S4 Table), as described in *S1 File*.

	**Description**	**k_{on}** (molecules^{-1} .µm^{2}.s^{-1})	**k_{off}** **(**s^{-1})	**K_D** (molecules. µm^{-2})	**Reference**
R1-R1	unligated VEGFR1 dimerization	8.0 x 10^{-4}	1.0 x 10^{-2}	12.5	See *S1 File*
R2-R2	unligated VEGFR2 dimerization	2.0 x 10^{-3}	1.0 x 10^{-2}	5	[5,6] and see *S1 File*
N1-R1	unligated NRP1-VEGFR1 dimerization	8.0 x 10^{-4}	1.0 x 10^{-2}	12.5	[16,26,34] and see *S1 File*
	**Description**	**k_{on}** **Surface** (molecules^{-1} .cell.s^{-1})	**k_{on}** **Rab4a** (molecules^{-1} .cell.s^{-1})	**k_{on}** **Rab11a** (molecules^{-1} .cell.s^{-1})	**Reference**
R1-R1	unligated VEGFR1 dimerization	8.0 x 10^{-7}	8.42 x 10^{-7}	2.46 x 10^{-6}	See *S1 File*
R2-R2	unligated VEGFR2 dimerization	2.0 x 10^{-6}	2.11 x 10^{-6}	6.15 x 10^{-6}	See *S1 File*
N1-R1	unligated NRP1-VEGFR1 dimerization	8.0 x 10^{-7}	8.42 x 10^{-7}	2.46 x 10^{-6}	See *S1 File*
