# Supplementary material for: Trafficking dynamics of VEGFR1, VEGFR2, and NRP1 in human endothelial cells
Source: PLoS Comput Biol. 2024 Feb 7;20(2):e1011798. doi: 10.1371/journal.pcbi.1011798 (PMC10878527; doi:10.1371/journal.pcbi.1011798)
Supplement: S4 Table — Includes sources for justification of key parameters from previous studies [25,41]. (PDF) [file pcbi.1011798.s023.pdf]

S4 Table. Other Model Parameters (for HUVECs)

Receptor species/ parameters	Value	Units	Reference
Cell membrane surface area	1000	μm^2	[41]
Rab4a/5a endosomes surface area	950	μm^2	see *S1 File*
Rab11a endosomes surface area	325	μm^2	see *S1 File*
VEGFR1 (cell surface)	1,800	receptors/cell	[25]
VEGFR2 (cell surface)	4,900	receptors/cell	[25]
NRP1 (cell surface)	68,000	receptors/cell	[25]
