# Supplementary material for: Trafficking dynamics of VEGFR1, VEGFR2, and NRP1 in human endothelial cells
Source: PLoS Comput Biol. 2024 Feb 7;20(2):e1011798. doi: 10.1371/journal.pcbi.1011798 (PMC10878527; doi:10.1371/journal.pcbi.1011798)
Supplement: S5 Table — Based on modeling and data from PAECs (Porcine Aortic Endothelial Cells) (2015–2017) [7,12]. Abbreviations: R1: VEGFR1, R2: VEGFR2, N1: Neuropilin-1. *assumed same as VEGFR2. Includes sources for justification of key parameters from previous studies [7,12,16,33,42,43] (PDF) [file pcbi.1011798.s024.pdf]

S5 Table. Previous unligated receptor trafficking parameters
Based on modeling and data from PAECs (Porcine Aortic Endothelial Cells) (2015-2017) [7,12]
Abbreviations: R1: VEGFR1, R2: VEGFR2, N1: Neuropilin-1. *assumed same as VEGFR2

Parameter type	Receptor	Values [references]	Units
Internalization k_{int}	R2	2.6×10^{-3} [16,33,42,43]	s^{-1}
	N1	2.6×10^{-3} [16,33,42,43]*	s^{-1}
	R1	2.6×10^{-3} [33]*	s^{-1}
Recycling to surface via Rab4a k_{rec4}	R2	3.8×10^{-3} [16]	s^{-1}
	N1	3.8×10^{-5} [16]	s^{-1}
	R1	3.8×10^{-3} [33]*	s^{-1}
Recycling to surface via Rab11a k_{rec11}	R2	1.4×10^{-4} [16]	s^{-1}
	N1	1.4×10^{-2} [16]	s^{-1}
	R1	1.4×10^{-4} [33]*	s^{-1}
Transfer from Rab4a to Rab11a k_{4to11}	R2	1.0×10^{-5} [16]	s^{-1}
	N1	1.9×10^{-2} [16]	s^{-1}
	R1	1.0×10^{-5} [33]*	s^{-1}
Degradation k_{deg}	R2	8.6×10^{-6} [16]	s^{-1}
	N1	1.6×10^{-4} [16]	s^{-1}
	R1	8.6×10^{-5} [33]	s^{-1}
Production k_{prod}	R2	0.28 [16]	$rec.cell^{-1}.s^{-1}$
	N1	3.5 [16]	$rec.cell^{-1}.s^{-1}$
	R1	0.1 [33]	$rec.cell^{-1}.s^{-1}$
