# Supplementary material for: Trafficking dynamics of VEGFR1, VEGFR2, and NRP1 in human endothelial cells
Source: PLoS Comput Biol. 2024 Feb 7;20(2):e1011798. doi: 10.1371/journal.pcbi.1011798 (PMC10878527; doi:10.1371/journal.pcbi.1011798)
Supplement: S6 Table — Based on modeling and data from HUVECs (Human Umbilical Vein Endothelial Cells) (2023). This is a statistical summary of 100 successful optimizations using different initial guesses. Abbreviations: R1: VEGFR1, R2: VEGFR2, N1: Neuropilin-1. (PDF) [file pcbi.1011798.s025.pdf]

S6 Table. New optimized unligated receptor trafficking parameters
Based on modeling and data from HUVECs (Human Umbilical Vein Endothelial Cells) (2023)
This is a statistical summary of 100 successful optimizations using different initial guesses.
Abbreviations: R1: VEGFR1, R2: VEGFR2, N1: Neuropilin-1.

Parameter type	Receptor	Mean	Median	σ (standard deviation)	Units	CV (coefficient of variation)
Internalization k_{int}	R2	2.4×10^{-4}	2.3×10^{-4}	2.0×10^{-5}	s^{-1}	0.08
	N1	3.1×10^{-4}	2.7×10^{-4}	2.7×10^{-4}	s^{-1}	0.87
	R1	2.3×10^{-2}	1.3×10^{-2}	2.3×10^{-2}	s^{-1}	1.03
Recycling to surface via Rab4a k_{rec4}	R2	4.6×10^{-6}	1.2×10^{-6}	7.1×10^{-6}	s^{-1}	1.54
	N1	3.9×10^{-2}	2.1×10^{-2}	3.7×10^{-2}	s^{-1}	0.95
	R1	1.7×10^{-3}	5.4×10^{-4}	2.7×10^{-3}	s^{-1}	1.62
Recycling to surface via Rab11a k_{rec11}	R2	7.8×10^{-2}	8.9×10^{-2}	2.6×10^{-2}	s^{-1}	0.33
	N1	6.5×10^{-3}	7.9×10^{-4}	2.1×10^{-2}	s^{-1}	3.32
	R1	9.5×10^{-2}	1.0×10^{-1}	1.3×10^{-2}	s^{-1}	0.14
Transfer from Rab4a to Rab11a k_{4to11}	R2	4.8×10^{-6}	1.5×10^{-6}	7.0×10^{-6}	s^{-1}	1.46
	N1	5.2×10^{-2}	7.0×10^{-2}	4.5×10^{-2}	s^{-1}	0.87
	R1	6.7×10^{-4}	5.9×10^{-4}	3.7×10^{-4}	s^{-1}	0.55
Degradation k_{deg}	R2	2.4×10^{-4}	2.3×10^{-4}	4.1×10^{-6}	s^{-1}	0.02
	N1	6.9×10^{-6}	1.2×10^{-6}	1.7×10^{-5}	s^{-1}	2.51
	R1	2.3×10^{-4}	2.3×10^{-4}	1.6×10^{-5}	s^{-1}	0.07
Production k_{prod}	R2	1.153	1.112	0.141	rec. $cell^{-1}.s^{-1}$	0.12
	N1	0.836	0.479	0.785	rec. $cell^{-1}.s^{-1}$	0.94
	R1	3.776	3.693	0.398	rec. $cell^{-1}.s^{-1}$	0.11
