# Supplementary material for: Trafficking dynamics of VEGFR1, VEGFR2, and NRP1 in human endothelial cells
Source: PLoS Comput Biol. 2024 Feb 7;20(2):e1011798. doi: 10.1371/journal.pcbi.1011798 (PMC10878527; doi:10.1371/journal.pcbi.1011798)
Supplement: S7 Table — (PDF) [file pcbi.1011798.s026.pdf]

S7 Table. List of experimental reagents and antibodies

Reagents	Company (catalog #)	
HUVECs	Lonza (#2519A)<br>Lot #s: 0000704189 and 0000661173	
HUVEC culture media and supplements	EBM-2 medium supplemented with the bullet kit (EGM-2) (Lonza)	
Cycloheximide (CHX)	Sigma (C7698)	
Chloroquine diphosphate (CHQ)	Sigma (C6628)	
siRNA Rab4 oligonucleotide	ThermoFisher 439084 (s11675)	
siRNA Rab11 oligonucleotide	ThermoFisher 4390824 (s16702)<br>or Santa Cruz (sc3630)	
siRNA transfection reagent Lipofectamine 3000	Thermo Fisher Scientific	
Biotinylation kit	Pierce Cell Surface Biotinylation<br>and Protein Isolation Kit (cat#A44390)	
**Antibody**	**Company**	**IB titer**
VEGFR1 (membrane-integral)	CST (#2893)	1:1000
VEGFR2 (membrane-integral)	CST (#2479)	1:10000
NRP1 (membrane-integral)	R&D (AF3870)	1:1000
α-Tubulin	CST (#3873)	1:100000
β-Actin	CST (# 3700)	1:10000
Rab4a	ThermoFisher (MA5-17161)	1:1000
Rab11a	Abcam (ab65200), BD Bio (610656)	1:2000
Anti-mouse IgG, HRP-linked (secondary)	R&D (AF3870)	1:1000
Anti-rabbit IgG, HRP-linked (secondary)	CST (#3873)	1:100000

Abbreviations
IB: Immunoblot, **CST:** Cell Signaling Technologies,
SCBT: Santa Cruz Biotechnology, **R&D:** R&D Systems
HUVECs: Human Umbilical Vein Endothelial Cells
VEGFR: Vascular Endothelial Growth Factor Receptor, **NRP:** Neuropilin
