# Supplementary material for: Trafficking dynamics of VEGFR1, VEGFR2, and NRP1 in human endothelial cells
Source: PLoS Comput Biol. 2024 Feb 7;20(2):e1011798. doi: 10.1371/journal.pcbi.1011798 (PMC10878527; doi:10.1371/journal.pcbi.1011798)
Supplement: S8 Table — (PDF) [file pcbi.1011798.s027.pdf]

S8 Table. Correlation metrics between initial parameter guesses and optimized parameter guesses, for the 100 optimized parameter sets (see also S18 Fig).

parameter	VEGFR1	VEGFR2	NRP1
k_{int}	0.022	0.104	-0.063
k_{deg}	-0.065	-0.104	-0.012
k_{rec4}	-0.043	0.029	0.506
k_{4to11}	0.269	-0.046	0.432
k_{rec11}	0.255	0.005	0.149
